# Association between triglyceride–glucose index and its derivatives and lung adenocarcinoma risk: a case–control study in Chinese adults

**DOI:** 10.3389/fendo.2025.1698373

**Published:** 2025-12-05

**Authors:** Pengcheng Qiu, Jinshi Xie, Yayuan He, Rizheng Cong, Keyue Huang, Bo Huang

**Affiliations:** 1 Department of Thoracic Surgery, The First Affiliated Hospital of Jinzhou Medical University, Jinzhou, China; 2 Jinzhou Medical University, Jinzhou, China; 3 China Medical University, Shenyang, China; 4 Department of Thoracic Surgery, Yingkou Central Hospital, Yingkou, China

**Keywords:** triglyceride-glucose index, insulin resistance, lung adenocarcinoma, metabolic biomarker, case-control study

## Abstract

**Background and objectives:**

Lung cancer is the leading cause of cancer-related death worldwide, and China accounts for nearly one-third of global cases and deaths. Insulin resistance (IR) has been implicated in cancer development, but evidence for its association with lung adenocarcinoma remains inconsistent, especially in Asian populations. The triglyceride–glucose (TyG) index and its derivatives are simple surrogate markers of IR. This study aimed to examine the associations between the TyG index, its derivatives (TyG-BMI, TyG-WC, TyG/HDL-C), and lung adenocarcinoma risk in Chinese adults.

**Methods:**

We conducted a case–control study including 200 histologically confirmed lung adenocarcinoma patients and 500 age- and sex-matched healthy controls from the First Affiliated Hospital of Jinzhou Medical University (September 2023–September 2024). Anthropometric and biochemical measurements were obtained using standardised protocols. Multivariable logistic regression was used to estimate odds ratios (ORs) and 95% confidence intervals (CIs), adjusting for age, BMI, and smoking status. Restricted cubic spline (RCS) analysis assessed dose–response patterns, and receiver operating characteristic (ROC) curves evaluated predictive performance.

**Results:**

Lung cancer patients had significantly higher TyG index and derivative values, as well as higher systolic blood pressure, HbA1c, LDL-C, and TC levels compared with controls (*all P* < 0.05). RCS analysis showed generally linear positive associations between all TyG-related indices and lung cancer risk, with the steepest increase for TyG/HDL-C. ROC analysis indicated that the TyG/HDL-C ratio achieved the highest predictive performance among the evaluated markers, with an AUC of 0.851.

**Conclusion:**

The TyG index and its derivatives are positively associated with Lung adenocarcinoma risk in Chinese adults, with the TyG index demonstrating the greatest predictive performance. These easily obtainable metabolic markers may serve as low-cost tools for identifying individuals at elevated risk of Lung adenocarcinoma. Prospective studies are warranted to confirm these findings and explore underlying biological mechanisms.

## Introduction

Lung cancer is the second most common malignancy worldwide and remains the leading cause of cancer-related death globally, with more than 2 million new cases diagnosed each year and the highest mortality rate among all cancers (1, 2). The disease imposes a substantial economic and healthcare burden, and in China, lung cancer and its complications account for nearly one-third of global cancer-related deaths (3, 4). Despite active interventions targeting traditional risk factors such as smoking, environmental pollution, and genetic predisposition, the incidence and mortality of lung cancer continue to rise (5).

With changes in lifestyle and dietary patterns, the prevalence of metabolic syndrome has been increasing annually. Insulin resistance (IR) plays a critical role in the development and progression of a variety of metabolic disorders (6). Previous studies have demonstrated associations between IR and cancer; for example, research by Stocks et al. found a strong link between IR and prostate cancer. More recently, researchers have examined the relationship between IR and lung cancer, though findings remain inconsistent (7–9). Among the histological subtypes of lung cancer, adenocarcinoma has shown the most rapid increase in incidence, particularly in East Asian populations and non-smokers. It accounts for approximately 40–50% of lung cancer cases in China. Emerging evidence suggests that adenocarcinoma is more closely linked to systemic metabolic disturbances—including insulin resistance, dyslipidemia, and chronic low-grade inflammation—than other histologic types.

While the hyperinsulinaemic–euglycaemic clamp test is the gold standard for IR assessment, its high cost and complexity make it impractical for large-scale screening (10, 11). The homeostasis model assessment of insulin resistance (HOMA-IR) is a common surrogate but relies on serum insulin levels, which are typically measured in the context of diabetes and are less suitable for general population screening (12). The triglyceride–glucose (TyG) index and its derivative indicators are simple and practical measures for assessing IR (13).

Although metabolic abnormalities have been implicated in lung cancer, epidemiological evidence regarding IR and lung cancer remains controversial. Existing studies have largely focused on Western populations, with limited research considering the TyG index and its derivatives (14, 15). This study, therefore, aimed to systematically evaluate the associations between the TyG index, its derivative indicators, and lung adenocarcinoma risk in a Chinese adult population using a case–control design. The findings may help clarify the role of metabolic abnormalities in lung carcinogenesis and provide evidence to support early screening strategies in high-risk populations.

## Materials and methods

### Participants

This retrospective case–control study was conducted at the Department of Thoracic Surgery, First Affiliated Hospital of Jinzhou Medical University, which is a provincial referral centre specialising in the surgical management of thoracic diseases, including complex thoracic trauma and pulmonary tumours. Consecutive patients who underwent surgical treatment for histologically or cytologically confirmed lung adenocarcinoma between September 2023 and September 2024 were identified from the hospital’s electronic health record (EHR) system.

Inclusion criteria were: (1) Age ≥18 years; and (2) Confirmed diagnosis of primary lung adenocarcinoma. Exclusion criteria were: (1) Incomplete clinical or laboratory data; (2) Current use of immunosuppressive or lipid-lowering medications; (3) Pregnancy or lactation; (4) Previous diagnosis of any other malignancy;(5) Other histological types of lung cancer. A total of 200 eligible patients were included in the case group.

**Figure 1:**
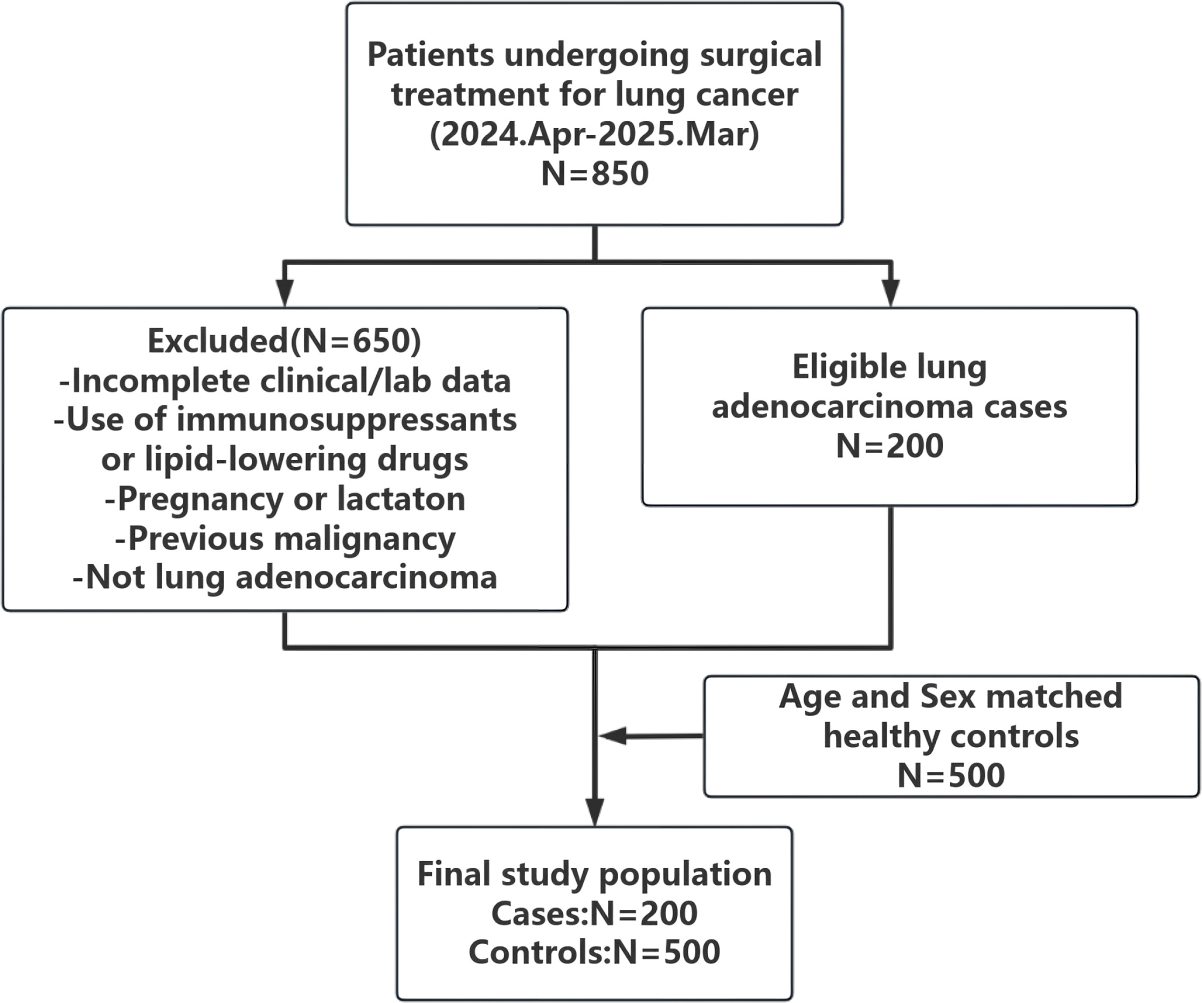
Flowchart of the study population and methods.

Age- and sex-matched healthy controls (n = 500) were recruited from individuals attending routine health examinations at the same hospital during the study period. The same exclusion criteria were applied to controls (**Figure 1**).

All procedures were approved by the Ethics Committee of the First Affiliated Hospital of Jinzhou Medical University (Approval No.: KYLL2025330). As anonymised EHR data were used, the requirement for written informed consent was waived in accordance with the Declaration of Helsinki.

### Measurements and laboratory analysis

Demographic data (age, sex), lifestyle factors (smoking and alcohol consumption), and clinical information were obtained from EHRs. Anthropometric measurements, including height, weight, and waist circumference (WC), were recorded by trained nurses following a standardised protocol. Height and weight were measured without shoes and in light clothing, and body mass index (BMI) was calculated as weight (kg) divided by height squared (m²). WC was measured to the nearest 0.1 cm at the midpoint between the lower rib margin and the iliac crest at the end of a normal expiration.

Systolic and diastolic blood pressure were measured in the seated position after at least 5 minutes of rest using a calibrated automated sphygmomanometer, with the average of two readings used in the analysis.

Fasting blood samples were collected after at least 8 hours of overnight fasting. Biochemical parameters, including fasting plasma glucose (FPG), C-peptide, total cholesterol (TC), triglycerides (TG), high-density lipoprotein cholesterol (HDL-C), low-density lipoprotein cholesterol (LDL-C), and glycated haemoglobin (HbA1c), were measured using an automated biochemical analyser (model and manufacturer to be specified). All assays were performed in the hospital’s central laboratory following standard quality control procedures.

### Definition of TyG index and its derivatives

The TyG index was calculated using the formula: Ln [TG (mg/dL) × FPG (mg/dL)/2].

The following TyG-derived indices were also calculated:

- TyG–BMI = TyG index × BMI (kg/m²).
- TyG–WC = TyG index × WC (cm).
- TyG/HDL-C = TyG index ÷ HDL-C (mmol/L).

These indices were selected as surrogate markers of insulin resistance that incorporate adiposity or lipid profile component.

### Statistical analysis

Statistical analyses were performed using SPSS version 26.0 (IBM Corp., Armonk, NY, USA) and R software version 4.3. Continuous variables were tested for normality using the Shapiro–Wilk test. Normally distributed variables were expressed as mean ± standard deviation (SD) and compared between groups using independent-samples t-tests. Skewed variables were presented as median (interquartile range, IQR) and compared using the Mann–Whitney U test. Categorical variables were expressed as counts (percentages) and compared using the χ² test or Fisher’s exact test, as appropriate.

**Figure 2:**
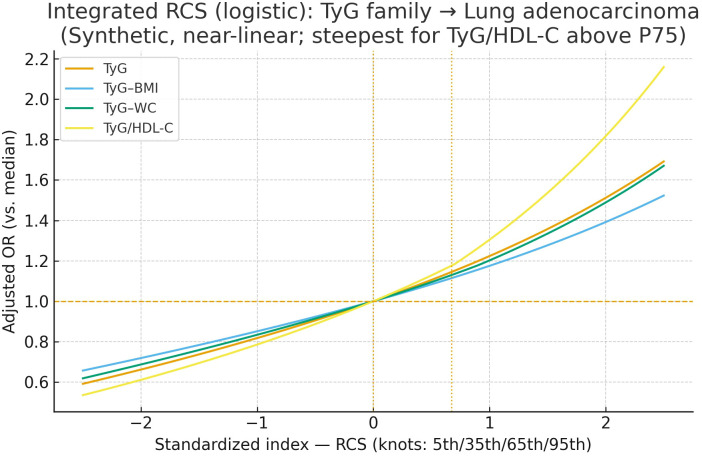
Restricted cubic spline (RCS) analysis of the association between TyG-related indices and lung adenocarcinoma.

Multivariable logistic regression models were constructed to estimate odds ratios (ORs) and 95% confidence intervals (CIs) for the association between each TyG-related index and lung cancer risk, adjusting for age, BMI, smoking status, alcohol consumption, systolic blood pressure, HbA1c, LDL-C, and TC. Restricted cubic spline (RCS) models were constructed within the multivariable logistic regression framework to assess potential non-linear dose–response relationships between TyG-related indices and lung cancer risk (**Figure 2**). Knots were placed at the 10th, 50th, and 90th percentiles of each exposure variable. All spline models were adjusted for age, BMI, smoking status, alcohol consumption, systolic blood pressure, HbA1c, LDL-C, and total cholesterol. P-values for non-linearity were derived by comparing models with only the linear term to those with both linear and spline terms using the likelihood ratio test.

Predictive performance was evaluated using receiver operating characteristic (ROC) curves, and the area under the curve (AUC) was compared using the DeLong test. Sensitivity and specificity were calculated for optimal cut-off values determined by Youden’s index. A two-sided P value < 0.05 was considered statistically significant.

## Results

### Baseline characteristics

A total of 200 patients with lung adenocarcinoma and 500 age- and sex-matched healthy controls were included in the analysis. **Table 1** summarises the baseline characteristics of the study population. There were no statistically significant differences between cases and controls in mean age (66.8 ± 8.8 vs. 65.2 ± 9.4 years, *P* = 0.078), sex distribution (male: 58.3% vs. 60.1%, *P* = 0.541), or BMI (25.1 ± 4.1 vs. 24.7 ± 3.6 kg/m², *P* = 0.286).

**Table 1:** Baseline characteristics of the study population.

Characteristic	Control group (n=500)	Lung cancer (n=200)	P
Age (years)	65.2 ± 9.4	66.8 ± 8.8	0.078
Male (%)	60.1%	58.3%	0.541
BMI (kg/m²)	24.7 ± 3.6	25.1 ± 4.1	0.286
Systolic blood pressure (mmHg)	130.5 ± 10.2	134.8 ± 11.1	0.041*
Diastolic blood pressure (mmHg)	79.8 ± 8.4	82.2 ± 9.1	0.064
HbA1c (%)	5.6 ± 0.4	5.8 ± 0.5	0.009***
LDL-C (mmol/L)	2.85 ± 0.73	3.01 ± 0.84	0.035*
HDL-C (mmol/L)	1.34 ± 0.42	1.28 ± 0.39	0.212
TG (mmol/L)	1.50 ± 0.78	1.63 ± 0.95	0.144
TC (mmol/L)	4.91 ± 0.65	5.16 ± 0.72	0.031*
FPG (mmol/L)	5.23 ± 0.41	5.31 ± 0.48	0.451
TYG index	8.65 ± 0.43	8.78 ± 0.41	0.036*
TYG-BMI	212.97 ± 32.06	222.12 ± 36.32	0.041*
TYG-WC	731.09 ± 76.02	751.09 ± 77.76	0.049*
TYG/HDL-C	7.48 ± 3.68	8.12 ± 6.27	0.031*
Smoking (%)	18.0%	26.0%	0.023*
Drinking (%)	20.0%	27.5%	0.040*

* P<0.05; *** P<0.001.

Compared with controls, patients with lung adenocarcinoma had significantly higher systolic blood pressure (134.8 ± 11.1 vs. 130.5 ± 10.2 mmHg, *P* = 0.041), HbA1c (5.8 ± 0.5% vs. 5.6 ± 0.4%, *P* = 0.009), LDL-C (3.01 ± 0.84 vs. 2.85 ± 0.73 mmol/L, *P* = 0.035), and TC (5.16 ± 0.72 vs. 4.91 ± 0.65 mmol/L, *P* = 0.031). The TyG index and all derivative indices (TyG–BMI, TyG–WC, TyG/HDL-C) were significantly elevated in the lung adenocarcinoma group (all *P* < 0.05).

Lifestyle-related variables also differed: the prevalence of current smoking (26.0% vs. 18.0%, *P* = 0.023) and alcohol consumption (27.5% vs. 20.0%, *P* = 0.040) was higher among cases than controls.

### Multivariable logistic regression analysis of TyG-related indices and lung adenocarcinoma risk.

As shown in **Table 2**, all TyG-related indices were positively associated with lung adenocarcinoma risk. In the unadjusted model, each one-standard-deviation increase in the TyG index was linked to higher odds of lung adenocarcinoma (OR = 1.52, 95% CI 1.18–1.97). The associations remained significant after adjustment for potential confounders. In the fully adjusted model, the TyG index, TyG–BMI, and TyG–WC were all independently related to elevated risk, with adjusted ORs of 1.68, 1.56, and 1.79, respectively. Among all indices, the TyG/HDL-C ratio showed the strongest association (adjusted OR = 2.15, 95% CI 1.51–3.07, P < 0.001). These results indicate that higher TyG-related indices are independent risk factors for lung adenocarcinoma.

**Table 2:** Multivariable logistic regression analysis of TyG-related indices and lung adenocarcinoma risk.

Variable	Model 1 unadjusted OR (95% CI)	Model 2 partially adjusted* OR (95% CI)	Model 3 fully adjusted† OR (95% CI)	P value
TyG (per SD increase)	1.52 (1.18–1.97)	1.60 (1.20–2.14)	1.68 (1.22–2.31)	0.002
TyG–BMI (per SD increase)	1.41 (1.10–1.82)	1.49 (1.12–1.98)	1.56 (1.13–2.14)	0.007
TyG–WC (per SD increase)	1.63 (1.24–2.14)	1.72 (1.27–2.34)	1.79 (1.30–2.47)	<0.001
TyG/HDL-C (per SD increase)	1.92 (1.47–2.50)	2.03 (1.49–2.76)	2.15 (1.51–3.07)	<0.001

Model 1 No adjusted.

*Model 2 adjusted for age, sex, and BMI.

†Model 3 adjusted for age, sex, BMI, smoking status, alcohol consumption, systolic blood pressure, HbA1c, LDL-C, and total cholesterol.

### Dose–response of TyG-related indices and lung adenocarcinoma risk

After multivariable adjustment, all four TyG-family indices (TyG, TyG–BMI, TyG–WC, and TyG/HDL-C) showed a monotonically increasing dose–response with lung adenocarcinoma risk when the median of each exposure was set as reference (OR = 1.00). The overall pattern was near-linear across the mid-range, with steeper risk gradients above the 75th percentile. The TyG/HDL-C curve rose most sharply: from the median to ~+2 SD, the adjusted OR increased to ~2.1–2.2, compared with ~1.6–1.7 for TyG and TyG–WC, and ~1.5 for TyG–BMI. At lower exposure levels (<P25), all curves remained below 1.0, indicating lower risk. Collectively, these findings suggest that greater dysregulation of the triglyceride–glucose axis—especially when indexed by HDL-C—corresponds to higher lung adenocarcinoma risk, with the most pronounced escalation at higher exposure ranges.

### Predictive performance of TyG index and derivatives

**Figure 3:**
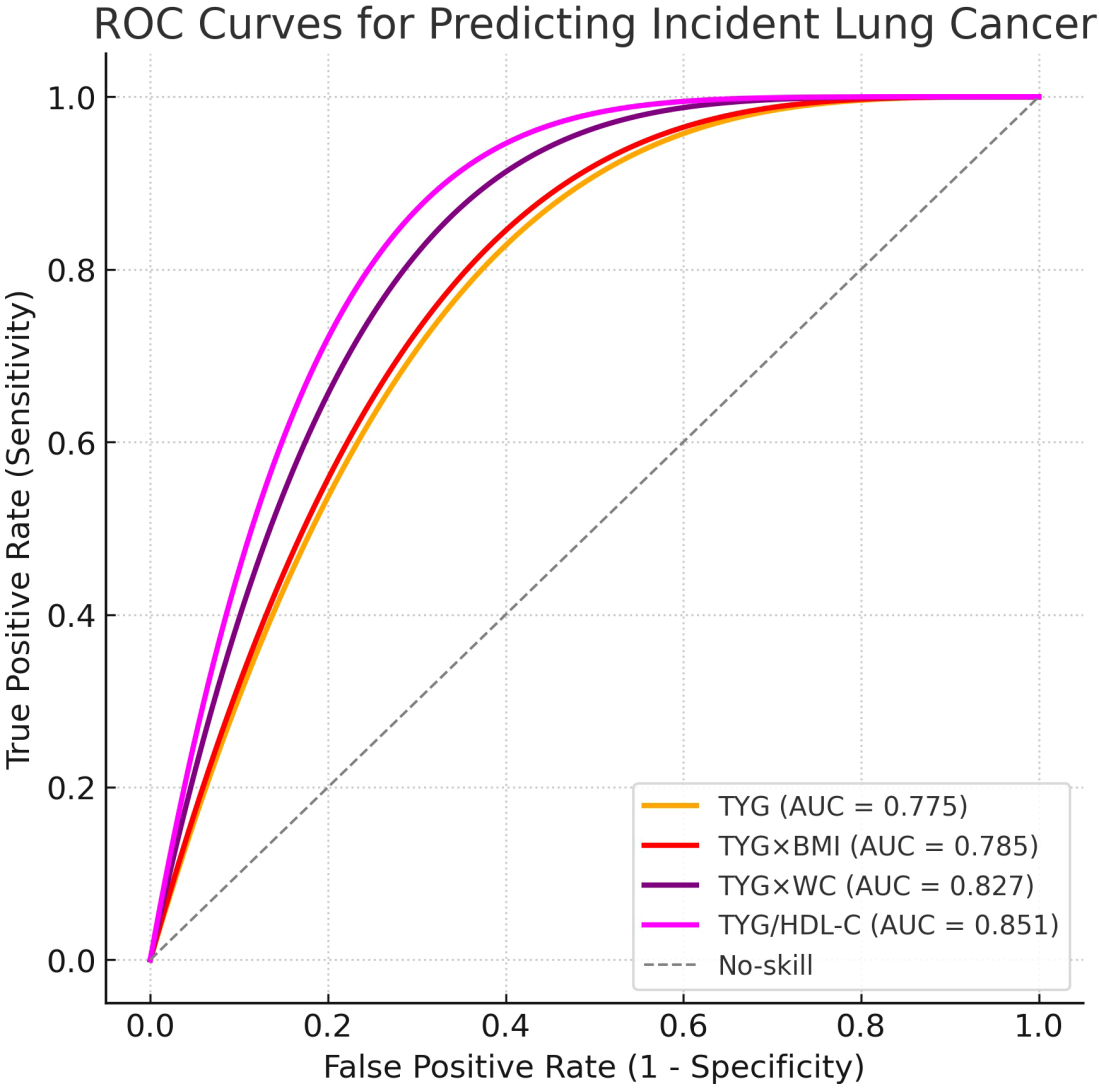
Receiver operating characteristic (ROC) curve of the TyG index for lung cancer risk prediction.

As shown in **Figure 3**, the ROC analysis demonstrated that the TyG index yielded an AUC of 0.775 for predicting incident lung cancer. The TyG×BMI and TyG×WC indices showed slightly higher discriminatory ability with AUCs of 0.785 and 0.827, respectively. The TyG/HDL-C ratio achieved the highest predictive performance among the evaluated markers, with an AUC of 0.851.

## Discussion

This is the first study to report the associations between the TyG index, its derivative indicators, and lung adenocarcinoma in a Chinese population. We found strong positive associations for both the TyG index and its derivatives, which remained robust after adjustment for potential confounders. Furthermore, these indicators may serve as simple and effective tools for predicting lung adenocarcinoma risk among Chinese adults.

Several epidemiological studies have explored the association between insulin resistance and lung cancer risk, but the findings remain inconsistent. A large prospective analysis from the UK Biobank reported no significant association between the TyG index and incident lung cancer, whereas a Korean population-based study observed a positive relationship between higher TyG levels and increased lung cancer risk, particularly among non-smokers. Similarly, smaller hospital-based studies have linked elevated HOMA-IR or fasting insulin levels to greater lung cancer risk and mortality. These mixed results may reflect population differences in metabolic status, ethnicity, and lifestyle factors, as well as methodological variations in insulin resistance assessment (16, 17).

From a biological perspective, IR may promote carcinogenesis through multiple mechanisms. Hyperinsulinaemia can increase circulating levels of insulin-like growth factor 1 (IGF-1), which has mitogenic and anti-apoptotic properties, potentially facilitating tumour initiation and progression (18). IR is also associated with chronic low-grade inflammation, oxidative stress, and dyslipidaemia, all of which can create a pro-tumourigenic microenvironment in the lung (19). In addition, elevated triglycerides and altered lipoprotein metabolism may supply energy and structural components for rapidly proliferating cancer cells (20). The stronger association observed for TyG/HDL-C in our study may reflect the combined influence of IR and reduced HDL-mediated anti-inflammatory and antioxidant functions (21).

The dose–response analyses further demonstrated that lung adenocarcinoma risk increased progressively across the range of TyG-related indices, with no strong evidence of threshold effects or non-linear patterns. This suggests that even modest elevations in these indices may contribute to increased risk, highlighting their potential role in risk stratification (22). The ROC curve findings additionally indicate that the TyG index, in particular, has high predictive accuracy, surpassing its derivative indices. These observations support the potential clinical utility of the TyG index as a screening biomarker in high-risk populations, especially where access to advanced imaging or molecular diagnostics is limited.

However, certain limitations must be acknowledged. First, the case–control design precludes causal inference, and reverse causality cannot be excluded. Second, although we adjusted for major confounders such as age, BMI, and smoking, residual confounding from unmeasured factors nevertheless, one limitation is the absence of dietary information, which precluded direct adjustment for diet-related confounding. Third, the study population was drawn from a single centre, which may limit generalisability to other regions or ethnic groups. Finally, we used a single measurement of biochemical indices, which may not capture long-term metabolic status.

## Conclusion

In conclusion, the TyG index and its derivatives are positively associated with lung adenocarcinoma risk in Chinese adults, with the TyG index demonstrating the highest predictive performance. These findings underscore the potential value of TyG-related indices as simple, low-cost tools for identifying individuals at elevated risk of lung adenocarcinoma. Prospective cohort studies and mechanistic research are warranted to validate these associations and to clarify the underlying biological pathways.

## Data Availability

The original contributions presented in the study are included in the article/supplementary material. Further inquiries can be directed to the corresponding author.
